# The social wasps as a reservoir of non-*Saccharomyces* yeasts for bio-protection strategies in winemaking

**DOI:** 10.1007/s00253-025-13636-6

**Published:** 2025-11-25

**Authors:** Damiano Barbato, Simona Guerrini, Viola Galli, Eleonora Mari, Marzia Cristiana Rosi, Lisa Granchi

**Affiliations:** 1 FoodMicroTeam S.R.L, Ex - Academic Spin-Off of the University of Florence, Via Santo Spirito 14, 50125 Florence, Italy; 2 Department of Agriculture, Food, Environment and Forestry (DAGRI), University of Florence, Via san Bonaventura 13, 50145 Florence, Italy

**Keywords:** Social wasps, Non-*Saccharomyces* yeasts, Bio-protection, Grapes, Winemaking, *Metschnikowia pulcherrima*

## Abstract

**Abstract:**

Recent studies on yeast-insect associations demonstrated that social wasps of the genera *Polistes* and *Vespula* act as a reservoir for the conservation of yeasts and as vectors capable of transferring such yeasts on the grapes. This work aimed to assess yeast species associated with social wasps and to obtain new strains to be used as bio-protection agents in winemaking. The wine yeast communities present on the exoskeleton, intestine of social wasps, and on the surface of grapes sampled in the vineyards of three Tuscan wineries were determined. Regardless of the wasp species, yeasts were mostly associated with female workers and found mainly in their intestine (up to 7 × 10^5^ CFU/mL). The identification revealed 20 species belonging to 10 genera; *Aureobasidium pullulans* and *Metschnikowia pulcherrima* were isolated from wasps of all the wineries, the latter occurring at the highest frequency. Strain-level characterization highlighted that three strains present on grapes were also present in the gut of wasps from the same vineyard. All the isolated *M. pulcherrima* strains underwent *in vitro* tests to select the most suitable for use as bio-protective cultures. Three strains showed good inhibitory activity against *Kloeckera apiculata* and *Brettanomyces bruxellensis*; hence, they were selected for bio-protection trials on artificially contaminated grapes. Results highlighted the reduction of non-*Saccharomyces* populations, suggesting the effectiveness of *M. pulcherrima* as a biocontrol agent. The study confirmed the role of social wasps as yeast vectors in the vineyard and as a reservoir of yeast strains to be exploited for biotechnological applications in oenology.

**Key points:**

• *Social wasps can carry yeast strains that are also present on the surface of grapes*

• *Metschnikowia pulcherrima was found within the wasp gut as the prevalent yeast species*

• *M. pulcherrima proved to be a suitable biocontrol agent on grapes*

**Supplementary Information:**

The online version contains supplementary material available at 10.1007/s00253-025-13636-6.

## Introduction

Non-*Saccharomyces* yeasts are endemic in vineyard environments, including soil and surfaces of vines and grapes, and, at harvest time, they usually belong to different genera like *Candida*, *Metschnikowia*, *Hanseniaspora*, *Cryptococcus*, and *Rhodotorula* (Barata et al. 2012). Nevertheless, the yeast community can change over time, depending on several factors, including the ripening stage, vineyard location, cultivar, and viticultural practices (Griggs et al. 2021). In addition, insects may disperse yeasts, with honeybees, social wasps, and *Drosophila* flies implicated as vectors (Goddard et al. 2010; Lam and Howell 2015; Stefanini et al. 2012). Studies of insect-driven yeast dispersal suggest that their activity is integral to shaping the seasonal assembly of the microbiome in plants (Madden et al. 2017, 2018). Yeasts predominantly colonize the insect digestive tract where they may act as nutrient providers, digestion facilitators, or protectors against pathogens and toxic compounds, proving to be crucial for insect development and survival (Malassigné et al. 2021; Stefanini 2018). Social wasps are very promising as potential yeast vectors; indeed, the *Vespa crabro* L. and *Polistes dominulus* queens, overwintering as adults, can carry yeast cells from autumn to spring and transmit them to their progeny, thus contributing to the survival of yeasts during the unfavourable season (Stefanini et al. 2012). Besides, Di Paola et al. (2023) highlighted the possibility of using *P. dominulus* as a biotechnological tool to directly spread yeasts (e.g., *Saccharomyces cerevisiae* strain) in the vineyard to control the sensory characteristics of the final product. Few studies have assessed the yeast community carried by these insects, and there is little information about their concentrations. Stefanini and colleagues (2012) analyzed the guts of 61 Hymenoptera (*V. crabro*, *Polistes* spp., and *Apis mellifera*) collected in Italy, finding *Candida* as the most represented genus (43% of the isolates), followed by *Pichia* spp. (32% of the isolates). The frequency of several species varied according to the season, while *S. cerevisiae*, which constituted 4% of the yeast gut community, showed minimal seasonal changes. Another work by Jimenez et al. (2017) investigated the gut of wasps from five species in the Pacific Northwest. Yeasts of the genera *Lachancea* and *Hanseniaspora* comprised 35% of the isolates, 25% were identified as *Metschnikowia* spp., and the remaining 10% belonged to *Rhodotorula*. In contrast to Stefanini et al. (2012), *S. cerevisiae* was not detected in any wasp species. Valentini et al. (2022) showed that the surrounding habitat, such as woodland, can influence the yeast populations carried by wasps into vineyards. However, certain yeast species, such as *Lachancea thermotolerans* and *Metschnikowia pulcherrima*, can be consistently found in the guts of wasps regardless of the environment. Currently, in oenology, interest in recovering new strains of non-*Saccharomyces* yeasts is increasing due to their potential ability to enhance wine aroma (Borren and Tian 2021) and their bioprotective activity (Comitini et al. 2023; Di Gianvito et al. 2022) as an alternative to the use of sulphites to protect musts, mainly from potential alteration by indigenous microbiota at the pre-fermentative steps (Di Canito et al. 2021). Among the non-*Saccharomyces* yeasts, *Metschnikowia pulcherrima* stands as one of the most promising species as a bioprotective agent and for improving the aroma of wine (Morata et al. 2019; Puyo et al. 2023). In fact, some commercial *M. pulcherrima* cultures for the bio-protection of grape are now available, such as Primaflora VB (AEB, San Polo (BS), Italy), LEVEL^2^ GUARDIA™ (Lallemand, Montreal, Canada), and ZYMAFLORE® KHIO (Laffort, Floirac, France). The bioprotective activity can be attributed to several mechanisms, as reviewed by Sipickzi (2020). A primary role is the iron immobilization by pulcherrimin production; but, although iron depletion is a robust inhibitory mechanism, other mechanisms can also contribute to the antagonistic activity of the *Metschnikowia* cells, such as nitrogen source competition and chitinase secretion (Puyo et al. 2023).

In the present work, to enhance the knowledge of yeast species associated with social wasps and to obtain new yeast strains to be used as bio-protection agents in winemaking, wasps were caught in the vineyards of three different wineries in Tuscany at the harvest, and the populations of yeasts occurring both on the exoskeleton and in the gut were quantified and identified. The yeasts isolated from wasps were compared to yeasts occurring on the grapes of the same vineyards where the wasps were captured. The *M. pulcherrima* isolates were then evaluated for their bio-protection potential, and the most effective ones were used to assess their impact on grapes intentionally contaminated with undesirable wine yeast species (*Hanseniaspora uvarum*, *Brettanomyces bruxellensis*, and *Starmerella bacillaris*) in a laboratory bio-protection trial.

## Materials and methods

### Insect and grape sampling

Insects and grapes were collected at the stage of technological grape maturity, immediately before harvest at three wineries in the Tuscany region (central Italy), each with a controlled designation of origin (DOCG) status: Chianti Classico, Brunello di Montalcino, and Vino Nobile di Montepulciano. The wineries were labelled as follows: MP (Villa Montepaldi—San Casciano in Val di Pesa, Florence), AR (Argiano srl Società Agricola—Montalcino, Siena), and AV (Avignonesi srl—Montepulciano, Siena).

Vineyards were selected based on similar features (pruned-spur cordon Sangiovese vineyard, with at least one side covered by spontaneous trees and shrub species). Wasps were collected over two consecutive days using an entomological net by moving along the inter-row of the vineyard, covering the entire area examined (vineyard areas were 1.2, 4.4, and 7.6 HA for MP, AR, and AV winery, respectively). Collections were performed during the warmest hours of the day, when wasps were more active (approximately between 10:00 AM and 15:00 PM). Insects were then placed into 50-mL sterile tubes and kept refrigerated until transferred to the laboratory. Once at the laboratory, autoclaved water–soaked cotton (121 °C for 20 min) was inserted into each tube to provide a water source, thereby preventing dehydration and enhancing wasp survival. Samples were stored at 4 °C until dissection, which occurred within 24 h of collection. Simultaneously with insect sampling, grapes were harvested by randomly collecting different bunches in sterile plastic bags (approximately 2 kg of total grapes per vineyard), which were stored under refrigerated conditions until the transfer to the laboratory.

### Yeast quantification on insects and grapes

Insects were euthanized according to the procedure described by Valentini et al. (2022), with slight modifications, by exposure at −21 °C for 15 min and then processed to collect microorganisms on the exoskeleton and guts. To determine microorganisms on the exoskeleton, each insect was placed in 1.5-mL Eppendorf tubes completely covered with a 300-μL sterile saline solution (9 g/L NaCl in distilled water) and sonicated (ARGOLAB DU-06, Sinergica Soluzioni S.r.l, Milan, Italy) for 2 min to promote the detachment of microorganisms. The obtained solution was plated on WL nutrient agar medium (Oxoid Ltd., Basingstoke, Hampshire, UK) integrated with sodium propionate (VWR International Srl, Milan, Italy) (2 g/L) and streptomycin (VWR International Srl, Milan, Italy) (30 mg/L) to inhibit mould and bacteria growth, respectively. The wasps were then externally sterilized by immersing specimens three times in succession in 70% ethanol, as described by Jimenez et al. (2017). Excess ethanol was removed using sterile absorbent paper, and the samples were rinsed in sterile water. Under sterile conditions and using a stereomicroscope, the wasps were then dissected in sterile Petri dishes with tweezers. The entire digestive tract, including the crop (foregut), midgut, and hindgut, was extracted and mechanically ground using a glass pestle and suspended in a 130-μL sterile saline solution and subjected to the pour plate method. Regarding grapes, 10 g of samples, transferred into 90 mL of sterile physiological saline solution (9 g/L NaCl), were homogenized for 2 min in a Stomacher Lab Blender 400 (Seward Ltd., Worthing, West Sussex, UK). Then, 100 μL of these suspensions were plated directly (1 mL) or after decimal dilutions on MYPG agar (5 g/L malt extract, 3 g/L yeast extract, 5 g/L meat extract, 10 g/L glucose, and 20 g/L agar) by using the pour plate method under sterile conditions. Plates were performed in duplicate and incubated for 48 h at 30 °C under aerobic conditions. The detection limit was 10 CFU/mL. After purification, the yeasts were grown on a YEPD (10 g/L of yeast extract, 20 g/L of peptone, and 20 g/L of dextrose) medium and maintained at −80 °C in a solution containing 50% (v/v) glycerol until identification.

### Identification and typing of yeasts

After purification of the colonies, the yeasts were grown on a YEPD medium. The isolates were identified through amplification of the 5.8S rDNA and the two ribosomal internal transcribed spacers (ITS) by using the primers ITS1 and ITS4 as described by Granchi et al. (1999). The obtained amplicons were digested using *Hae*III, *Cfo*I, *Hinf*I, and *Dra*I as restriction enzymes (Life Technologies Italia, Monza, Italy). Yeast cells picked from 24-h-old colonies were suspended in 50-µL sterile water, and then 2 µL were directly used for all PCR reactions.

To confirm the identification, the D1/D2 domain of the 26S rDNA gene was amplified, and the PCR products were purified using Nucleo Spin Extract II (Macherey–Nagel GmbH & Co. KG, Düren, Germany) according to the manufacturer’s instructions before sending them to BMR Genomics (Padua, Italy) for sequencing. The sequences obtained in FASTA format were compared to sequences available in the GenBank database (http://www.ncbi.nlm.nih.gov/) using the basic BLAST search tools.

*M. pulcherrima* isolates were typed by randomly amplified polymorphic DNA (RAPD) analysis performed using the primer M13 (5′-GAGGGTGGCGGTTCT-3′) (Huey and Hall 1989) as reported by Barbosa et al. (2018) and the PCR protocol according to Reguant and Bordons (2003). All reactions included both negative (DNA-free) and positive controls, and the PCR was processed in an Applied Biosystems® 2720 Thermal Cycler (Life Technologies, Monza, Italy). Reproducibility of RAPD–PCR patterns was assessed by comparing the PCR products obtained with DNA prepared from two separate cultures of the same strains.

### In vitroantimicrobial activity screening of *M. pulcherrima *strains

Intra- and extracellular pulcherrimin production was evaluated according to Sipiczki (2022) with some modifications. Yeast cultures were grown for 24 h at 30 °C in YEPD medium. The cultures were spotted on agar plates containing YEPD supplemented with 0.005 g/100 mL of FeCl_3_. After 72 h at 25 °C, colonies producing pulcherrimin turn red on this medium. If pulcherrimin is also produced extracellularly, a colored halo can be seen around the pigmented colony. The pigmentation intensity was estimated by using a four-level scale: − = no production, + = weak production, + + = production, and + + = intense production. The halo development was evaluated as follows: − halo absence, + small halo, + + halo, + + + large halo. The inhibitory action of the strains showing higher pulcherrimin production was further investigated against some potential wine spoilage microorganisms (three strains of *Brettanomyces bruxellensis* and two strains of *Kloeckera apiculata*, belonging to the culture collection of the Department of Agriculture, Food, Environment and Forestry of the University of Florence, Italy), as reported by Oro et al. (2014)*.* The yeasts were grown for 24–72 h, respectively, at 30 °C in YEPD medium. Approximately 10^5^ CFU/mL of the cultures were uniformly suspended and poured into sterile 20 mL YEPD agar, supplemented with 0.005 g/100 mL of FeCl_3_. After solidification, *M. pulcherrima* suspensions (20 µL) were spotted on plates. The plates were incubated at 25 °C for 3–4 days. After this time, the color of the *M. pulcherrima* spots and the presence of inhibition halos against each sensitive strain were visually assessed and evaluated. The commercial starter *M. pulcherrima* LEVEL^2^ GUARDIA™ (Lallemand, Montreal, Canada) was also assayed as a control.

### Killer activity and toxin sensitivity of *M. pulcherrima* strains

The killer activity of *M. pulcherrima* strains was tested following Philliskirk and Young (1975), using the reference sensitive strain *S. cerevisiae* (NCYC 1006; National Collection of Yeast Cultures, Norwich, UK). *M. pulcherrima* strain was scored as a killer when the streaked strain was surrounded by a clear zone where no growth of the sensitive strain had occurred, bounded by a dark blue zone of dead cells. The sensitivity of *M. pulcherrima* strains to killer activity was tested by streaking the killer reference strain *S. cerevisiae* NCYC 738 (National Collection of Yeast Cultures, Norwich, UK) onto plates containing the *M. pulcherrima* strain to be tested, incorporated into the agar medium at approximately 10^5^ CFU/ml. The *M. pulcherrima* strain was deemed sensitive if it did not grow around the killer reference strain *S. cerevisiae* NCYC 738. Neutral *M. pulcherrima* strains neither produced nor were affected by the toxin.

### Biocontrol activity of *M. pulcherrima* strains on grapes and musts

The strains of *M. pulcherrima* with the highest antagonistic activity in vitro were also tested for their antagonistic activity on grapes artificially contaminated with undesirable wine yeast species and on the resulting must after grape pressing. Briefly, white grapes were purchased from a local supermarket and washed by immersion in a 2% sodium hypochlorite solution for 2 min. They were then rinsed in distilled water and dried at room temperature to disinfect the surface. A suspension of mixed cultures of *Starmerella bacillaris* (10^5^ CFU/g), *K. apiculata* (10^5^ CFU/g), and *B. bruxellensis* (10^2^ CFU/g) was prepared. Grapes were dipped in the suspension for 2 min and then dried under laminar flow for 2 h. Following the same protocol, the selected indigenous *M. pulcherrima* strains and the commercial strain LEVEL^2^ GUARDIA™ (Lallemand, Montreal, Canada) were applied as axenic cultures (10^7^ CFU/mL) on contaminated grapes to evaluate the bio-protection effect. Moreover, a negative control without *M. pulcherrima* was prepared. The fruits were packed in commercial plastic boxes and stored at 18 °C for 15 h. Then, grapes were pressed to obtain must subjected to 7 days of cryomaceration at 6 °C. Each yeast population was quantified by using the pour plate method after 15 h and 7 days of cryomaceration at 6 °C. The test was carried out in duplicate.

### Statistical analysis

The experimental data on yeast counts were analyzed for normality and homogeneity of variance, followed by one-way ANOVA and subsequent Tukey’s test or Holm-Šídák test (for multiple groups) using GraphPad Prism 8 software package (San Diego, CA, USA). A *p*-value less than 0.05 was considered significant*.* In addition, the obtained RAPD-PCR profiles corresponding to the different *M. pulcherrima* strains were subjected to unweighted pair group method using arithmetic averages (UPGMA) clustering analysis using the band-based Dice similarity coefficient and GelCompar 6.6 software (Applied Maths, St-Martens-Latem, Belgium).

## Results

### Quantification and identification of wasps and yeasts occurring on the exoskeleton and in the gut of the wasps

In the three Sangiovese vineyards considered, 57 wasps were captured. The wasp’s identification, carried out by observing their morphological characteristics (Dvořák and Roberts 2006; Schmid-Egger et al. 2017), revealed the presence of three species, *Polistes dominulus* (15.8%), *Polistes gallicus* (31.6%), and *Vespula germanica* (52.6%), which were distributed differently according to the winery (Table 1). In the vineyards of winery AR, only *V. germanica* occurred, while in the vineyards of winery AV, all three wasp species were found. Seventy-four percent of the total wasps carried yeasts: 59% belonged to the *V. germanica* species, followed by the *P. gallicus* and *P. dominulus* species at 24% and 17%, respectively (Table S1). Regardless of the wasp species, yeasts were mostly associated with female workers.

**Table 1:** Wasps’ species captured in Sangiovese vineyards of the three wineries (MP, AR, and AV)

Winery	N° of wasps	Species	Female worker	Male	Wasps carrying yeasts	
					**Female worker**	**Male**
MP	2	*Polistes dominulus*	2	0	1	0
	13	*Polistes gallicus*	7	6	5	2
AR	20	*Vespula germanica*	20	0	19	0
AV	7	*Polistes dominulus*	6	1	5	1
	5	*Polistes gallicus*	2	3	2	0 1
	10	*Vespula germanica*	10	0	6	0

**Fig. 1:**
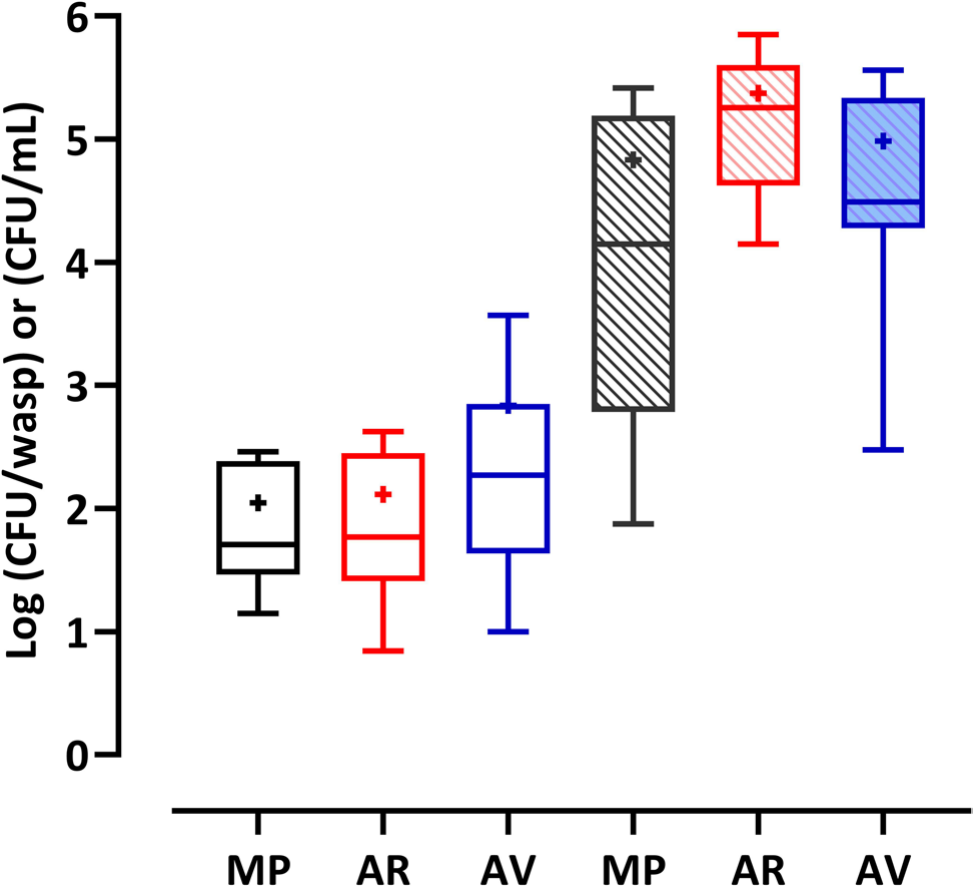
Box plots representing the yeast cell concentrations occurring on the exoskeleton (clear box) and in the gut (filled box) of wasps captured in Sangiovese vineyards of the three wineries (MP, AR, and AV). The center line of each box represents the median, the mean is indicated by “ + ”, the top and bottom of the box represent the 75th and 25th percentile, respectively (one-way ANOVA and Tukey’s test *p* < 0.05)

In addition, yeasts were found in 67% of wasps in the gut, 19% on the exoskeleton and in the gut, and 14% only on the exoskeleton (Table S1). Yeast cell concentrations in the gut of wasps, ranging from 7 × 10 to 7 × 10^5^ CFU/mL, were, on average, higher than cell amounts on the exoskeleton; nevertheless, in both cases, no significant differences were found among data from the three wineries (Fig. 1).

After quantifying the yeast populations associated with the wasps, the yeast isolates were identified by the PCR–RFLP of the ITS region, and the results are reported in Table S2. Yeast identification revealed 20 different species belonging to 10 genera (*Aureobasidium*, *Candida*,* Cyberlindnera*, *Kloeckera*, *Lachancea*, *Metschnikowia*, *Saccharomyces*, *Saccharomycopsis*, *Torulaspora*, and *Wickerhamomyces*) included in the *phylum Ascomycota*. In detail, 15 yeast species, including *S. cerevisiae*, were associated with 26 *V. germanica* wasps (the five yeast species that did not occur were *A. melanogenum*, *C. stellata*, *C. agrestis*, *C. sake*, and *L. thermotolerans*). Eight species (*A. pullulans*, *A. melanogenum*, *C. stellata*, *M. pulcherrima*, *K. apiculata*, *C. diversa*, *C. agrestis*, and *C. sake*) were associated with nine *P. gallicus* wasps, and five (*A. pullulans*, *M. pulcherrima*, *K. apiculata*, *C. diversa*, and *L. thermotolerans*) with seven *P. dominulus* wasps. Thirty-eight percent of the wasps carrying yeasts harboured a single yeast species. The distribution of yeast species based on their frequency in the wasps captured at the three wineries and the number of different strains of each species are shown in Table 2. Only the yeast-like fungus *Aureobasidium pullulans* and *Metschnikowia pulcherrima* were isolated from wasps of all the wineries. Moreover, *M. pulcherrima* occurred at the highest frequency, regardless of the winery, whereas *S. cerevisiae* was found just in one *V. germanica* gut wasp captured in the vineyards of the AR winery. *S. cerevisiae* was scarcely present inside the gut of wasps; indeed, it was found only in one wasp.

**Table 2:** Yeast species associated with social wasps, their frequency and number of yeast strains occurring on the exoskeleton (*E*) and in the gut (*G*) (MP, AR, and AV indicate the wineries), in bold species of oenological importance

Winery	Yeast species	Frequency (%)	N° strains (*E*)	N° strains (*G*)
MP	*Aureobasidium melanogenum*	16	3	0
	*Aureobasidium pullulans*	18	3	1*
	*Candida stellata*	23	0	1
	***Kloeckera apiculata***	18	0	3
	***Metschnikowia pulcherrima***	25	0	5
AR	*Aureobasidium pullulans*	5	1	3
	*Candida moniliforme*	1	0	1
	*Candida railenensis*	3	1	0
	*Candida wickerhamii*	1	0	2
	*Cyberlindnera americana*	1	0	1
	***Metschnikowia pulcherrima***	70	3	17^§^
	***Kloeckera apiculata***	5	0	5
	*Pichia anomala*	2	0	2
	*Pichia kluyveri*	5	0	2
	*Pichia rhodanensis*	3	2	0
	***Saccharomyces cerevisiae***	1	0	1
	***Torulaspora. delbrueckii***	1	0	1
	*Wickerhamomyces chambardii*	2	0	1
AV	*Aureobasidium melanogenum*	3	0	2
	*Aureobasidium pullulans*	30	4	5
	*Candida agrestis*	7	0	1
	*Candida diversa*	4	2	0
	*Candida railenensis*	2	1	0
	*Candida sake*	6	0	1
	***Metschnikowia pulcherrima***	43	1	8*
	*Saccharomycopsis capsularis*	3	1	0
	***Lachancea thermotolerans***	2	0	1

^*^1 strain occurred on both the exoskeleton and gut

^§^2 strains occurred on both the exoskeleton and gut

The characterization of yeast isolates at the strain level revealed that 11 out of 20 species were each represented by a single strain (Table 2), which was carried only by one wasp on the exoskeleton or in the gut. Independent of the yeast species, the wasps from the vineyards of the three wineries did not share any strains. Nevertheless, in a few wasps from the same vineyard, some common *M. pulcherrima* strains were found (data not shown).

### Quantification and identification of yeasts occurring on Sangiovese grapes

In the vineyards of the three wineries, at harvest time, simultaneously with the wasp capture, Sangiovese grapes were sampled to quantify, identify, and characterize at the strain level the occurring yeast populations.

Yeast concentrations were about 1.5 × 10^4^ CFU/mL for the AR and AV winery’s grapes and 1 × 10^5^ CFU/mL for the grapes of the MP winery. As expected, non-*Saccharomyces* yeasts were predominant and belonged to *Aureobasidium* spp., *K. apiculata*, and *M. pulcherrima*, whereas *S. cerevisiae* was found only at 4% on the grapes of the MP winery (Table 3). The characterization at the strain level pointed out that on the MP winery grapes, two strains (one of *K. apiculata* and one of *M. pulcherrima*) were also present in the gut of one wasp, *P. gallicus*, caught in the vineyard of the same winery. Likewise, one *K. apiculata* strain found on the AR winery grape also occurred in the gut of one *V. germanica* wasp from the same vineyard.

**Table 3:** Yeast species, their frequency, and number of yeast strains occurring on Sangiovese grapes sampled in vineyards of the three wineries (MP, AR, and AV), in bold species of oenological importance

Winery	Yeast species	Frequency (%)	N° strains
MP	*A. melanogenum*	6	1
	***K. apiculata***	80	1^*^
	***M. pulcherrima***	10	1^*^
	***S. cerevisiae***	4	1
AR	***M. pulcherrima***	77	3
	***K. apiculata***	23	1^§^
AV	*A. pullulans*	57	2
	***K. apiculata***	43	2

^*^Strains also present in the gut of one wasp captured in MP vineyard

^§^Strain also present in the gut of one wasp captured in AR vineyard

### Genotypic and phenotypic characterization of *M. pulcherrima *isolates

**Fig. 2:**
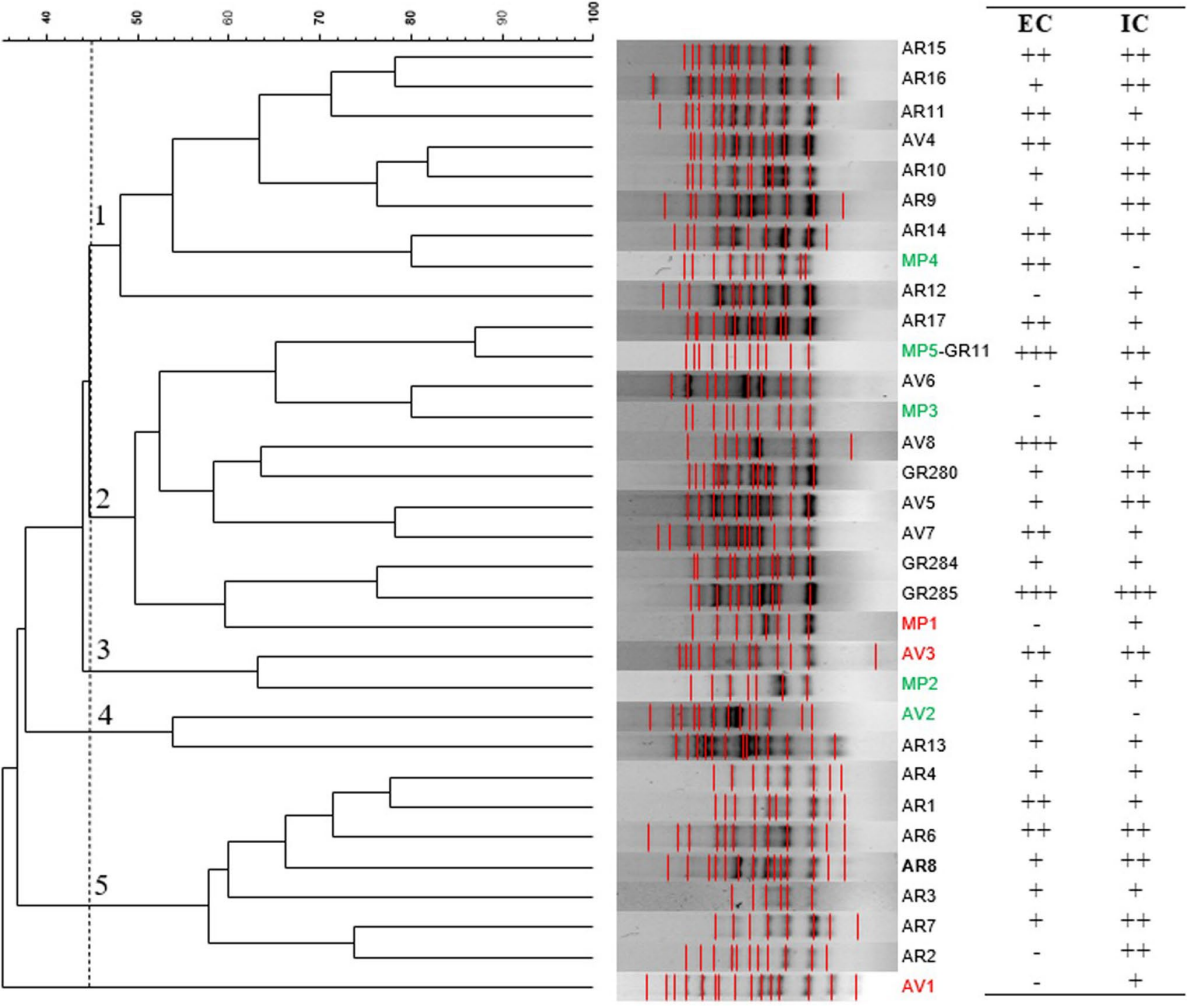
Dendrogram obtained from UPGMA analysis, using Dice’s coefficient, based on M13 random amplified polymorphic DNA (RAPD) profiles of *M. pulcherrima* strains isolated from wasps and grapes (GR) in three wineries (MP, AR, and AV) and, on the right, their pulcherrimin production by in vitro assay (EC, extracellular halo; −, halo absence; +, small halo; + +, halo; + + +, large halo; IC, intracellular production; −, no production; +, weak production; + +, production; and + +, intense production) (black, red, and green indicate *M. pulcherrima* strains from *V. germanica*, *P. dominulus*, and *P. gallicus* wasps, respectively)

*M. pulcherrima* was the yeast species associated with wasps at the highest percentage, regardless of the vineyard of origin. Moreover, it was also the predominant yeast species on grapes of the AR and AV companies. Because of the great interest in using this species for the bio-protection of grape and must, all the *M. pulcherrima* isolates (158) were genotypically characterized, and 30 different strains were found in total (Table S3). One or two predominant strains, showing frequency ranging from 21 to 36%, occurred in the population of *M. pulcherrima* detected in the wasps caught in the vineyards of each winery. Only one *M. pulcherrima* strain was also found on grapes in the vineyard of the MP winery. To evaluate the similarity among *M. pulcherrima* strains, all the different RAPD-PCR profiles (including those isolated from grapes) were subjected to UPGMA clustering analysis using the Dice coefficient. At a similarity threshold of 45%, the *M. pulcherrima* strains were grouped into five distinct clusters (Fig. 2). Clusters 1 and 5 predominantly contained strains isolated from wasps found in one winery vineyard, AR, indicating a significant similarity linked to their source of isolation. In contrast, clusters 2, 3, and 4 comprised *M. pulcherrima* strains derived from wasps collected in the vineyards of the MP and AV wineries, as well as from grapes.

To evaluate the bioprotective properties of the *M. pulcherrima* strains, the ability to produce the pigment “pulcherrimin” intracellularly and/or extracellularly was first determined by estimating the pigmentation intensity and the halo diameter around the colony, respectively. As shown in Fig. 2, differences among the strains were observed, with strains MP5-GR11 and -GR285 identified as the highest producers of “pulcherrimin.”

Regarding killer activity and sensitivity to killer toxin, all the tested *M. pulcherrima* strains were neutral, neither producing nor being affected by the toxin.

### Antimicrobial activity of *M. pulcherrima* strains: in vitro test

The *M. pulcherrima* strains exhibiting higher pulcherrimin production (GR285, MP5, AV8, AR6, AR14) were subsequently tested to assess their antimicrobial activity against two strains of *Kloeckera apiculata* (Ka1 and Ka2) and three strains of *Brettanomyces bruxellensis* (Bb1, Bb2, and Bb3), which are two yeast species that could negatively affect the final wine quality. The commercial *M. pulcherrima* strain LEVEL^2^ GUARDIA™ was used as a control. Three of the selected *M. pulcherrima* strains (GR285, MP5, AV8) significantly inhibited the growth of the *K. apiculata* Ka1 strain; the other two *M. pulcherrima* strains exhibited moderate activity, while the commercial strain showed no effect on the apiculate strains (Table 4). Concerning *B. bruxellensis*, strain-dependent sensitivity was shown; only the *M. pulcherrima* AR14 strain was able to inhibit the growth of the three *B. bruxellensis* strains tested, while the commercial strain showed poor activity against *B. bruxellensis*. Consequently, wild wasp–derived strains demonstrated a greater ability to inhibit spoilage yeasts compared to the commercial strain.

**Table 4:** Antimicrobial activity of selected *M. pulcherrima* strains and the commercial *M. pulcherrima* strain (LG) against strains of *K. apiculata* (Ka1, Ka2) and *B. bruxellensis* (Bb1, Bb2, Bb3) (− no activity, + low, + + medium, + + + high activity)

Non-*Sacch*	Strains of* M. pulcherrima*					
	**GR285**	**MP5**	**AV8**	**AR6**	**AR14**	**LG**
**Ka1**	$++$	+ +	$++$	$+-$	$+-$	$-$
**Ka2**	$-$	$-$	$-$	$-$	$-$	$-$
**Bb1**	$+-$	+	+	$-$	+ +	$+-$
**Bb2**	+	$+-$	$-$	$-$	+ +	$-$
**Bb3**	+	+	+	$+-$	+	$+-$

### Antimicrobial activity of M. pulcherrima strains: bio-protection trials

**Fig. 3:**
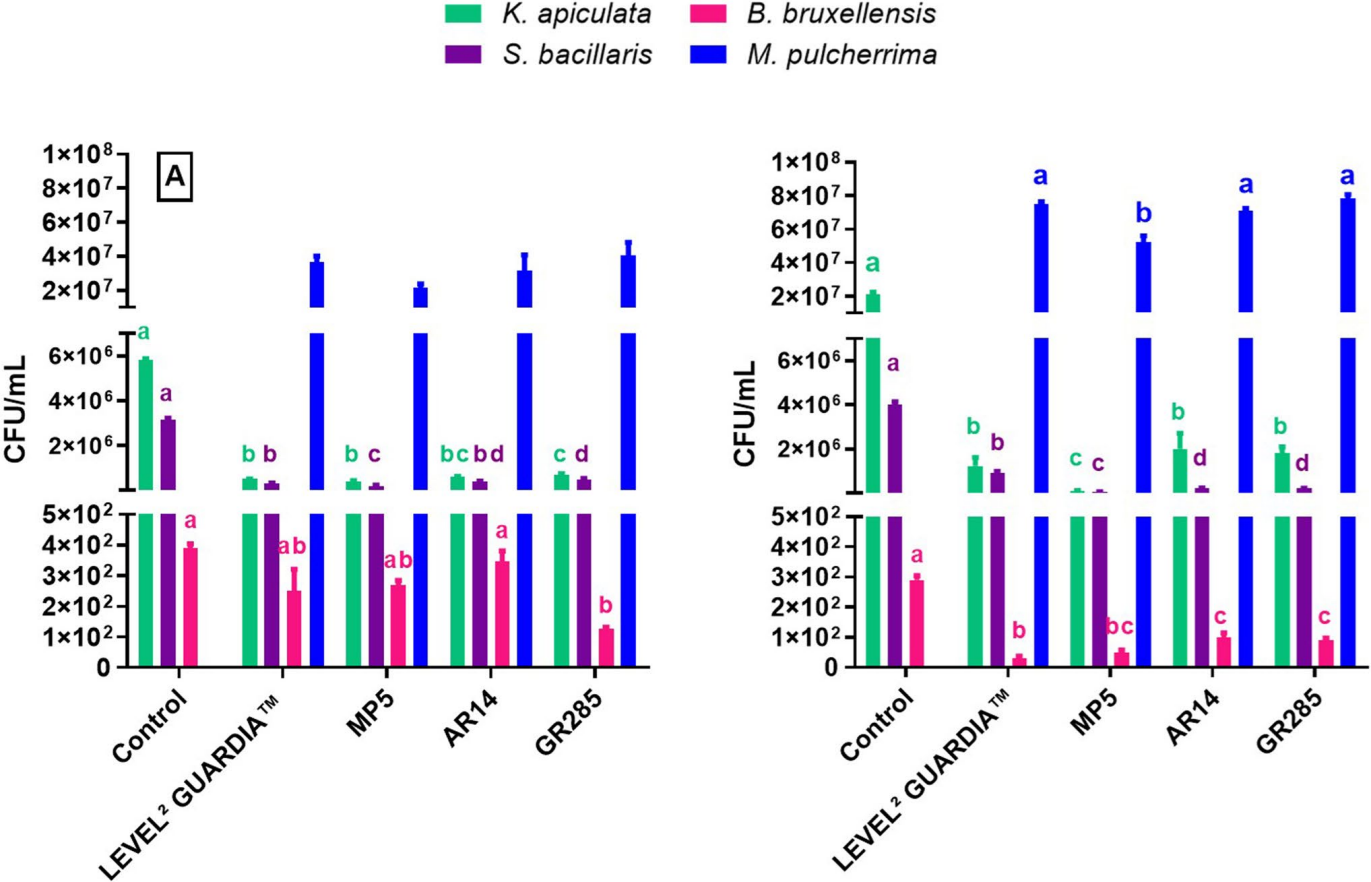
Yeast populations (CFU/mL) occurring in must after artificially contamination of grapes with mixed strains of *S. bacillaris*, *K*. *apiculata*, and *B. bruxellensis* after being treated with the selected *M. pulcherrima* strains (MP5, AR14, and GR285) and the commercial LEVEL^2^ GUARDIA™ strain after 15 h at 18 °C (**A**) and after 7 days of cryomaceration at 6 °C (**B**). Different letters indicate statistically significant differences (ANOVA and Holm-Šídák Test, *p* < 0.05) among the same yeast population

To test the actual bio-protection capabilities of the *M. pulcherrima* strains obtained from wasps, the selected strains (GR285, AR14, and MP5) were applied as axenic cultures (10^7^ CFU/mL) on the grapes previously contaminated with mixed strains of *S. bacillaris*, *K. apiculata*, and *B. bruxellensis*. The same test was conducted using the commercial *M. pulcherrima* strain LEVEL^2^ GUARDIA™. The grapes were then pressed and microbiologically analyzed after 15 h at 18 °C and after 7 days of cryomaceration at 6 °C to evaluate the bio-protection effect (Fig. 3).

All the *M. pulcherrima* strains significantly reduced the presence of *K. apiculata* and *S. bacillaris* on the grapes or during cryomaceration of at least one logarithmic unit. The strain that demonstrated the highest reduction capacity against *K*.* apiculata* and *S. bacillaris* (reduction percentage higher than 90%) in both conditions was the strain MP5. Finally, the reduction of *B. bruxellensis* was less significant on grape samples than during cryomaceration in the presence of all the *M. pulcherrima* strains tested, except in the presence of the GR285 strain, which determined a reduction percentage of about 70%. During cryomaceration, the commercial LEVEL^2^ GUARDIA™ and MP5 strains were more effective against *B. bruxellensis*, yielding both reduction percentages higher than 80%.

## Discussion

The current study aimed to evaluate social wasps as a reservoir of non-*Saccharomyces* yeasts for use in bio-protection strategies in winemaking. For this purpose, 57 wasps were captured at grape harvest in vineyards from three wineries in Tuscany (Italy), and yeast populations occurring on their exoskeleton and in their gut were quantified and identified at species and strain levels. Concurrently, in the same vineyards, ripe grapes were collected for the enumeration and characterization of the occurring yeasts.

The 57 wasps belonged to three species: *P. dominulus* (15.8%), *P. gallicus* (31.6%), and *V. germanica* (52.6%). Although the sample size was small, the frequency of individuals captured for the different species is consistent with seasonal patterns at grape harvest: individuals of the *V. germanica* are more numerous than those of the genus *Polistes*, and yeast communities and abundance are greater in *V. germanica* than in *Polistes* species (Valentini et al. 2022). In this context, our findings further support the pivotal role of social wasps as yeast vectors in the environment, as shown by other surveys (Stefanini et al. 2012; Valentini et al. 2022; Di Paola et al. 2023; Turillazzi et al. 2023). In addition, the results revealed that yeasts were found more frequently in females of all species, in agreement with Valentini et al. (2022), suggesting a significant difference between male and female wasps in their ability to serve as yeast vectors. This disparity may depend on their different roles within the colony. Indeed, female workers undertake various tasks, such as foraging and caring for larvae, during which yeast transmission can occur. In contrast, males primarily focus on reproduction and generally do not engage in significant social roles (Turillazzi 2003).

Quantification using culture-dependent methods of yeast populations associated with wasps revealed that cell concentrations were higher in the gut than on the exoskeleton of wasps, regardless of wasp species and the vineyard where they were collected. These findings confirm that the guts of social wasps carry yeasts that may survive throughout the winter and reproduce within this environment, as demonstrated by various studies (Stefanini et al. 2012; Jimenez et al. 2017; Madden et al. 2018; Valentini et al. 2022; Di Paola et al. 2023; Turillazzi et al. 2023). Furthermore, *V. germanica* hosted more yeast cells than *Polistes* spp., as noted by Valentini et al. (2022). This is likely due to *V. germanica*’s ability to form large underground colonies, which provide additional microbial sources, in contrast to *Polistes* spp. (Yurkov 2018; Ramírez et al. 2020; Turillazzi, 2003).

The identification of yeasts showed that the yeast-like fungus *A. pullulans* and *M. pulcherrima* were isolated from wasps caught in vineyards across all the wineries, and they occurred at high frequencies. These two yeast species have also been found in other investigations involving gut samples of social wasps (Valentini et al. 2022). *A. pullulans* has also been detected in the nest envelopes of *Vespula* species, where its hyphae appear to reinforce the nest structure (Durrell 1965) and was able to emit volatile compounds that attract wasps, suggesting a potential mutualistic relationship (Davis et al. 2012). Similarly, *Metschnikowia pulcherrima* is commonly found in floral nectar, indicating mutualism between plants and pollinators (Álvarez-Pérez et al. 2016, 2019). In addition, *M. pulcherrima* produces volatile semiochemicals such as acetic acid and isobutanol, which attract social wasps and are often used in baits to capture them (Sipiczki 2020; Landolt et al. 2000; Jimenez et al. 2017). Wasps of the *Polistes* and *Vespula* genera, which feed on sugars and flowers (Krenn et al. 2005), may act as the primary vectors for this yeast, also in vineyards. Among the other identified yeasts, *K. apiculata*, *L. thermotolerans*, *T. delbrueckii*, and *S. cerevisiae* were of oenological interest and occurred at low frequencies apart from *K. apiculata*. In contrast to the findings of Stefanini (2012, 2016), who reported that *S. cerevisiae* was present in 4% of the examined wasps, this study found it only in the gut of a single wasp. The presence of *L. thermotolerans* was also described by Jimenez et al. (2017) as the predominant species in the intestine of eusocial wasps from the Pacific Northwest.

Regarding the quantification and composition of yeast populations on grape berries at the harvest, the results aligned with findings from several studies (Barata et al. 2012; Loureiro et al. 2012; Pinto et al. 2014; Stefanini and Cavalieri 2018). The yeast communities were, indeed, primarily composed of *K. apiculata*, *A. pullulans*, and *M. pulcherrima*, while *S. cerevisiae* was confirmed to be absent or scarcely present on healthy grapes, according to Mortimer and Polsinelli (1999), reporting that, before maturation, grapes are almost free of *S. cerevisiae* (∼0.05%).

The characterization of yeast species at the strain level revealed that the wasps captured in each vineyard did not share any common strains. In addition, two strains (one of *K. apiculata* and one of *M. pulcherrima*) found in the gut of a *P. gallicus* wasp were also present on the grapes of the same vineyard where the wasp was caught. Similarly, a *K. apiculata* strain found in the gut of a *V. germanica* from a vineyard of another winery was likewise present on the grapes of the same vineyard. Finally, for *M. pulcherrima* strains isolated from wasps found in the AR winery vineyards, the cluster analysis demonstrated a significant similarity linked to their source of isolation. These findings suggest that social wasps carry yeast communities that might be specific to the local wine production area, suggesting the concept of *terroir*, as demonstrated by Di Paola et al. (2023). Additionally, they indicate that social wasps can contribute to the dispersal of yeasts in vineyards by actively feeding on grapes and breaking the skin to access the sugars, supporting the “dispersal–encounter hypothesis” proposed by Madden et al. (2018), whereby yeasts are dispersed by insects between spatially distant sugar resources, and, in turn, insects benefit from the yeasts that, producing volatile compounds, indicate the presence of these sugar resources.

In this study, *M. pulcherrima* was found in the social wasps caught in the three vineyards at frequencies of 25, 43, and 70% of the total yeast populations. Given its well-known bioprotective properties (Oro et al. 2014; Morata et al. 2019; Sipiczki 2020; Puyo et al. 2023), all isolated strains of *M. pulcherrima* underwent in vitro tests (ability to produce killer toxin, pulcherrimine, and inhibition of undesirable wine yeasts) to select the most suitable strains for use as Bio Protective Cultures (BPC). Three strains (GR285, AR14, and MP5) showed good inhibitory activity against *K. apiculata* and *B. bruxellensis*, two yeast species undesirable in winemaking. Our results indicate that they did not produce a killer toxin; therefore, the observed activity may be attributed to the formation of pulcherrimin as an iron chelator, confirming that this is the main mechanism of bioprotection in *M. pulcherrima* (Oro et al. 2014; Sipiczki 2020; Puyo et al. 2023). The effectiveness of the three *M. pulcherrima* strains was also demonstrated through bio-protection trials conducted at a laboratory scale on artificially contaminated grapes, simulating the presence of non-*Saccharomyces* yeast species that could negatively impact wine quality. The experimental trials highlighted the reduction of non-*Saccharomyces* growth both after 15 h at 18 °C and after 7 days of cryomaceration at 6 °C, suggesting that *M. pulcherrima* might be used as a biocontrol agent in the prefermentative stages of winemaking, as reported by Canonico et al. (2023). It should be noted that the strains isolated from wasps demonstrated significantly greater bioprotective ability in both in vitro and in vivo tests compared to the commercial strain used in this study, highlighting the importance of using new sources of wild yeasts.

## Conclusions

This study corroborated the fundamental role of social wasps, overall female workers, as yeast vectors in the environment. Indeed, social wasps have been demonstrated to carry yeast strains that are also present on the surface of grapes, thereby contributing to the development of yeast communities specific to the local wine production area. Although only three yeast strains were common between grapes and wasps, this finding is significant, as very few studies have demonstrated it. Social wasps may serve as a natural reservoir of biodiversity, providing a source for selecting yeast strains suitable for biotechnological applications. Particularly, selected *M. pulcherrima* strains could be a useful tool as biocontrol agents contributing to the reduction of sulphur dioxide in winemaking. Considering that some strains of *M. pulcherrima* may have enzymatic activities that enhance the aroma of wines (Canonico et al. 2023), this yeast species could be a valuable tool in winemaking. It could serve the dual purpose of improving the wine’s aromatic profile and counteracting the deterioration caused by the undesirable microflora in the wine. Indeed, it has recently been proposed that feeding wasps with selected fungal strains of oenological interest over an extended period could lead to modelling of the insect’s fungal microbiota according to the desired applicative interests (Di Paola et al. 2023).

## Supplementary Information

Below is the link to the electronic supplementary material.

ESM 1 (DOCX 16.5 KB)

## Data Availability

No datasets were generated or analysed during the current study.
