# Supplementary material for: The social wasps as a reservoir of non-Saccharomyces yeasts for bio-protection strategies in winemaking
Source: Appl Microbiol Biotechnol. 2025 Nov 25;109:263. doi: 10.1007/s00253-025-13636-6 (PMC12696146; doi:10.1007/s00253-025-13636-6)
Supplement: Supplementary file 1 [file 253_2025_13636_MOESM1_ESM.docx]

**Supplementary Tables**

**Table S1.** Wasps caught in three vineyards and wasps carrying yeasts (E = exoskeleton, G = gut).

| **Wasp species** | **Number of wasps caught** | **Wasps carrying yeasts** | **E** | **G** | **E+G** |
| --- | --- | --- | --- | --- | --- |
| *Polistes dominulus* | 9 | 7 | 2 | 5 | 0 |
| *Polistes gallicus* | 18 | 10 | 2 | 5 | 3 |
| *Vespula germanica* | 30 | 25 | 2 | 18 | 5 |
| Total | 57 | 42 | 6 | 28 | 8 |

**Table S2** Restriction analysis of the 5.8-ITS rDNA region and yeast species

| **ITS**  **(pb)** | ***Hinf*I**  **(pb)** | ***Hae* III**  **(pb)** | ***Dra*I**  **(pb)** | ***Cfo*I**  **(pb)** | **Specie** |
| --- | --- | --- | --- | --- | --- |
| 590 | 450-140 | - | - | 65-95-185-165 | *Aureobasidium melanogenum* |
| 580 | 290-170-120 | 450-150 | - | - | *Aureobasidium pullulans* |
| 400 | 280-170 | 350-100 | - | 210-190 | *Candida agrestis* |
| 450 | 230-220 | 450 | - | - | *Candida diversa* |
| 610 | 310-300 | 300-310 | - | - | *Candida railenensis* |
| 490 |  | 420-70 | - | - | *Candida sake* |
| 460 | 250-110 | 460 | - | - | *Candida stellata* |
| 640 | 320-320 | 640 | - | 640 | *Candida wickerhamii* |
| 510 | 320-190 | 510 | - | - | *Cutaneothreosporum moniliforme* |
| 620 | 300-200-120 | 550-70 | - | - | *Cyberlindnera americana* |
| 750 | 330-180 -150-70 |  | 420-280 | - | *Kloeckera apiculata* |
| 680 | 340-340 | 310-220-100 | - | 360-300 | *Lachancea thermotolerans* |
| 380 | 200-180 | 280-180 | - | - | *Metschnikowia pulcherrima* |
| 600 | 300-300 | 600 | - | 615 | *Pichia anomala* |
| 450 | 250-200 | 370-80 | - | - | *Pichia kluyveri* |
| 600 | 300-300 | 500-100 | - | - | *Pichia rhodanensis* |
| 850 | 360-350-120 | 320-225-180-145 | - | - | *Saccharomyces cerevisiae* |
| 670 | 335-335 | 500-100-70 | - | 600 | *Saccharomycopsis capsularis* |
| 800 | 410-380 | - | 345-315-133 | - | *Torulaspora delbrueckii* |
| 550 | 300-180-70 | 550 | - | - | *Wickerhamomyces chambardii* |

**Table S3**. *M. pulcherrima* strains from social wasps caught in the three wineries (MP, AR and AV) and their frequency. (Predominant strains are underlined)

| ***M. pulcherrima* strains** | **N. Isolates** | **Frequency (%)** |
| --- | --- | --- |
| MP1 | 2 | 18.2 |
| MP2 | 4 | 36.4 |
| MP3 | 1 | 9.1 |
| MP4 | 3 | 27.3 |
| MP5* | 1 | 9.1 |
|  |  |  |
| AR1 | 36 | 33 |
| AR2 | 4 | 3.7 |
| AR3 | 2 | 1.8 |
| AR4 | 2 | 1.8 |
| AR5 | 13 | 11.9 |
| AR6 | 18 | 16.5 |
| AR7 | 1 | 0.9 |
| AR8 | 14 | 12.8 |
| AR9 | 1 | 0.9 |
| AR10 | 2 | 1.8 |
| AR11 | 3 | 2.8 |
| AR12 | 4 | 3.7 |
| AR13 | 2 | 1.8 |
| AR14 | 3 | 2.8 |
| AR15 | 1 | 0.9 |
| AR16 | 1 | 0.9 |
| AR17 | 2 | 1.8 |
|  |  |  |
| AV1 | 2 | 5.3 |
| AV2 | 3 | 7.9 |
| AV3 | 7 | 18.4 |
| AV4 | 12 | 31.6 |
| AV5 | 8 | 21.1 |
| AV6 | 2 | 5.3 |
| AV7 | 3 | 7.9 |
| AV8 | 1 | 2.6 |

* = strain also present on grape in MP vineyard
